# Editorial: Bladder preservation options for bladder cancer

**DOI:** 10.3389/fonc.2024.1493854

**Published:** 2024-09-26

**Authors:** Nancy B. Davis, Deepak Kilari, Elizabeth R. Kessler, Woodson W. Smelser

**Affiliations:** ^1^ Merck & Co., Inc., Rahway, NJ, United States; ^2^ Division of Hematology and Oncology, Medical College of Wisconsin, Milwaukee, WI, United States; ^3^ Division of Medical Oncology, University of Colorado Anschutz Medical Campus, Aurora, CO, United States; ^4^ Division of Urologic Surgery, School of Medicine, University of Washington, Seattle, WA, United States

**Keywords:** bladder preservation, bladder cancer, trimodality therapy, bladder sparing, chemoradiation

The treatment paradigm of bladder cancer improved dramatically in the past decade, with both immunotherapy (IO) and antibody drug conjugates (ADC) resulting in longer durability of responses and survival, particularly in metastatic urothelial cancer (mUC). Recently, cisplatin-based chemotherapy was supplanted as first-line mUC treatment by IO/ADC therapy (1) where available and, where it is not, by platinum-based chemotherapy + IO (2). In muscle-invasive bladder cancer (MIBC), use of cisplatin-based neoadjuvant therapy (NAT) prior to radical cystectomy (RC) is standard of care (SOC) for those eligible to receive it. In cisplatin-ineligible (3) but surgically fit patients, RC alone, often followed by adjuvant chemotherapy or nivolumab (4) for high-risk residual disease, is pursued. Current trials are exploring both IO and ADCs in combination with, or in lieu of, cisplatin in the perioperative setting. If successful, these regimens have potential to transform peri-operative management for all MIBC patients, not just those ineligible for cisplatin.

Historically, bladder preservation, or trimodal therapy (TMT), was a treatment option for MIBC patients unfit or unwilling to undergo RC, and has evolved to include maximal transurethral resection of the bladder tumor (TURBT) followed by concurrent chemoradiation. Flexibility in radiosensitizing chemotherapy and radiation (RT) techniques maximize safety but are beyond the scope of this discussion. While there are no successful prospective randomized studies comparing RC to TMT, multiple retrospective analyses concluded that outcomes are similar as regards disease-specific and overall survival (OS) (5, 6). The recent phase 3 SPARE trial attempted to determine non-inferiority in OS of patients randomized to selective bladder preservation versus RC following NAT. The trial closed early for poor accrual, concluding “… patient preferences for treatments impacted willingness to undergo randomisation and acceptance of treatment allocation”, amid frequent non-compliance with randomized treatment strategy, with 24% of those randomized to RC actually receiving RT (7). Two phase 3 studies, SWOG1806 (NCT03775265) and KEYNOTE-992 (NCT04241185), are currently exploring the addition of IO to TMT vs TMT alone, while another, SunRise-2 (NCT04658862), is evaluating a novel intravesical chemotherapy-eluting device plus IO vs TMT alone; however, results from all are pending.

As patients increasingly question the need for definitive local therapy, particularly RC with short and long term morbidity, mortality and quality of life implications (8, 9), and as more evidence arises that not all patients require consolidative therapy following NAT (10), largely due to newly developed more active systemic regimens, there is renewed interest in developing trials centered on bladder preservation. These studies employ a composite clinical complete response (cCR) endpoint, as assessed by the following: cystoscopy, TURBT, MRI and ctDNA (11). These strategies focus on patients eligible, but willing to forego, RC after NAT, with observation or continued systemic therapy for those achieving cCR, and RC or TMT for those who do not. It should be noted that cCR requires absence of disease by *all* measures, with ctDNA continuing to be explored as a predictive marker for response to therapy. This combination of highly effective systemic therapies and promising biomarkers is driving the field to increasingly consider bladder preservation.

Bladder preservation has wide-reaching applicability. While mostly applied in MIBC for predominant urothelial histology, it has relevance in non-muscle invasive bladder cancer (NMIBC) also. Cystectomy is recommended in NMIBC for presence of lympho-vascular invasion and/or variant histology (12), high-risk T1 disease (13) and for BCG-unresponsive or BCG-relapsing disease (14). Despite a high risk of progression to MIBC, patients may be reluctant to undergo organ sacrifice for a non-invasive disease with minimal metastatic potential. Bladder sparing for variant histology MIBC is not commonly utilized, except for small cell bladder cancer where the use of chemotherapy with subsequent RC or TMT is extrapolated from small cell lung cancer (SCLC). Other pure variants of bladder cancer are usually treated with RC alone; notwithstanding high recurrence rates, there is a paucity of data regarding relative chemo- or radio-resistance (15) to support this.

The articles within this Research Topic highlight the wide-ranging utility of bladder preservation. Narayan et al., discuss the novel mechanism of action for nadofaragene firadenovec-vncg, an intravesical gene therapy approved for high-risk BCG-unresponsive NMIBC with carcinoma *in situ* (CIS) with or without papillary tumors, where SOC is RC due to a high risk of progression to MIBC or mUC, to prolong bladder preservation for patients ineligible for, or refusing, RC. Xiao et al., retrospectively analyzed the clinical efficacy, as measured by survival, of partial versus radical cystectomy in T2N0M0 sarcomatoid MIBC patients at a single institution, grounded on NCCN guidelines recommending partial cystectomy as one option for unifocal, clinical stage T2 MIBC, without concomitant CIS, amenable to segmental resection with adequate margins in appropriately selected patients. The association between radiation and prognosis in small cell bladder cancer after bladder-sparing surgery, either TURBT or partial cystectomy, was assessed in a population-based retrospective cohort study using the SEER database by Liang et al. by evaluating if the addition of radiation, or chemoradiation, improves cancer-specific survival versus no radiation, given that SOC for small cell bladder cancer commonly involves chemotherapy plus either surgery or radiation therapy, as extrapolated from SCLC. Finally, as the NCCN guidelines recommend multi-modality therapy, without offering Category 1 endorsements, the retrospective analysis by Garg et al., utilized the National Cancer Database to compare overall survival of NAT plus RC or TMT or systemic therapy in patients with cT1-4aN2/3M0 MIBC, a group of patients historically considered suboptimal for TMT.

Treatment of urothelial cancer requires both a multidisciplinary team to achieve optimal clinical outcomes and employment of shared decision-making (16) based on the patient’s preferences and values, either of which may be hindered by a myriad of intrinsic or extrinsic factors. As bladder preservation request increase, the bladder cancer community needs to diligently define the tradeoffs of all options within the context of each individual patient’s case. All (wo)men are created equal … not all cancers are. Entering the next decade of discovery, optimism abounds for novel therapeutics, innovative endpoints and efficacy measures, and escalation of patient perspectives to maximize positive outcomes while minimizing unnecessary loss of organ or organ function.
